# Case report of misdiagnosis: a rare case of hepatic epithelioid hemangioendothelioma characterized primarily by fever

**DOI:** 10.3389/fonc.2025.1606872

**Published:** 2025-09-01

**Authors:** Jian Zhao, Yanli Dang

**Affiliations:** ^1^ Department of Hepatobiliary Surgery, Yunnan University Affiliated Hospital, Kunming, China; ^2^ Department of Obstetrics, The First People’s Hospital of Yunnan Province, Kunming, China

**Keywords:** hepatic epithelioid hemangioendothelioma, liver inflammatory myofibroblastic tumor, misdiagnosis, case report, fever

## Abstract

This study reports a case of a 47-year-old female who was initially misdiagnosed as having liver abscess due to repeated fever, and was later misdiagnosed again as liver inflammatory myofibroblastic tumor(IMT) after liver biopsy. After surgical removal, molecular pathological examination confirmed the diagnosis of Hepatic epithelioid hemangioendothelioma(HEHE). HEHE with fever as the main symptom is extremely rare and there are no similar reports available at present. This study summarizes the key points of HEHE diagnosis and treatment, analyzes the reasons for the misdiagnosis in this case and the possible factors causing the fever symptoms, highlights the difficulties in HEHE diagnosis and the significant value of molecular pathology in its diagnosis.

## Introduction

Hepatic epithelioid hemangioendothelioma (HEHE) is a rare borderline tumor originating from vascular endothelial cells. The etiology of HEHE is still unclear. Molecular pathological studies have revealed that WWTR1-CAMTA1 gene fusion caused by chromosomal translocation plays a key role in the pathogenesis of HEHE. The clinical manifestations of HEHE are significantly heterogeneous. Due to the lack of typical symptoms, the clinical manifestations are often overlooked and lead to misdiagnosis. This article reports the diagnosis and treatment of a patient with fever as the main manifestation and finally diagnosed as HEHE. The analysis of this rare case aims to provide diagnostic and therapeutic insights for future studies.

## Case report

**Figure 1:**
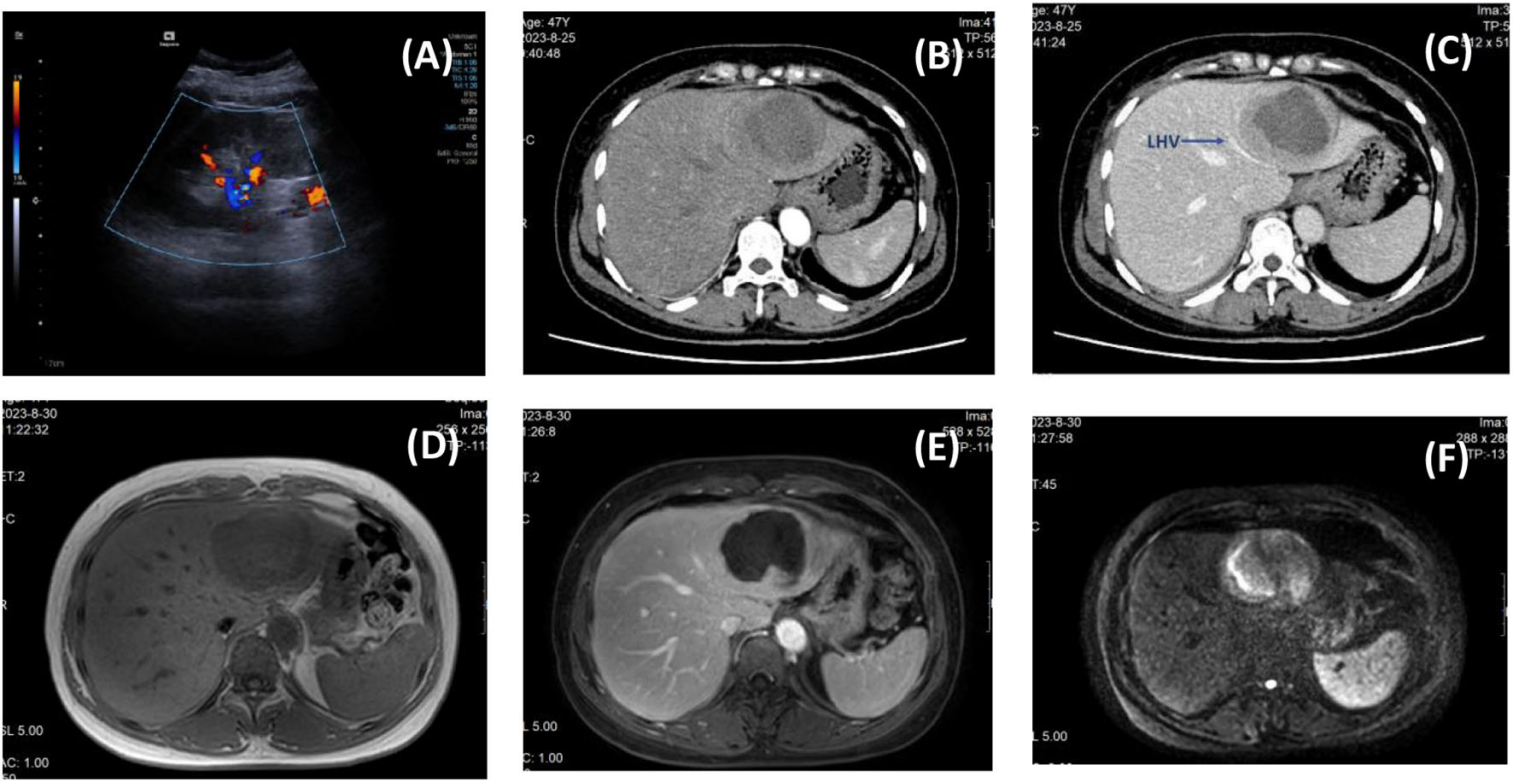
Preoperative imaging findings. [**(A)** B-ultrasound; **(B)** Arterial phase of enhanced CT; **(C)** Portal phase of enhanced CT, the blue arrow indicates the compressed left hepatic vein; **(D)** Fat imaging in MRI; **(E)** Arterial phase of MRI; **(F)** DWI image in MRI.].

A 47-year-old woman was admitted with recurrent high fever for 4 months, with a maximum body temperature of 39.6 °C. Physical examination was unremarkable. Laboratory tests showed: N% 78.6%, PLT 405×109/l, eosinophil count 0.01×109/l, CD3+/CD4+T cells count 304.53 cells/ul, ESR 95mm/h, CRP 260.6mg/l, PCT 0.016ng/ml, Lactic acid 4.70mmol/L, IL-2 19.6µg/L, IL-6 210.3µg/L, and other indicators such as tumor markers, EB/respiratory associated virus/hepatitis virus markers, G test, blood bacterial culture, autoimmune disease antibody, Vidal test, complement binding test, T cell test for tuberculosis infection were all negative. B-ultrasonography revealed a large hypoechoic mass in the left liver with mixed internal flow signals (**Figure 1A**). The enhanced CT scan revealed a central low-density mass and the left hepatic vein being compressed and terminating at the edge of the lesion — similar to the “lollipop sign” (**Figures 1B, C**). Contrast-enhanced MRI showed central hypointensity and peripheral hyperintensity (but not the typical “target sign”) (**Figure 1D**), and partial depression of the liver capsule — “liver capsule retraction sign” (**Figure 1E**). Diffusion was limited on DWI (**Figure 1F**).

**Figure 2:**
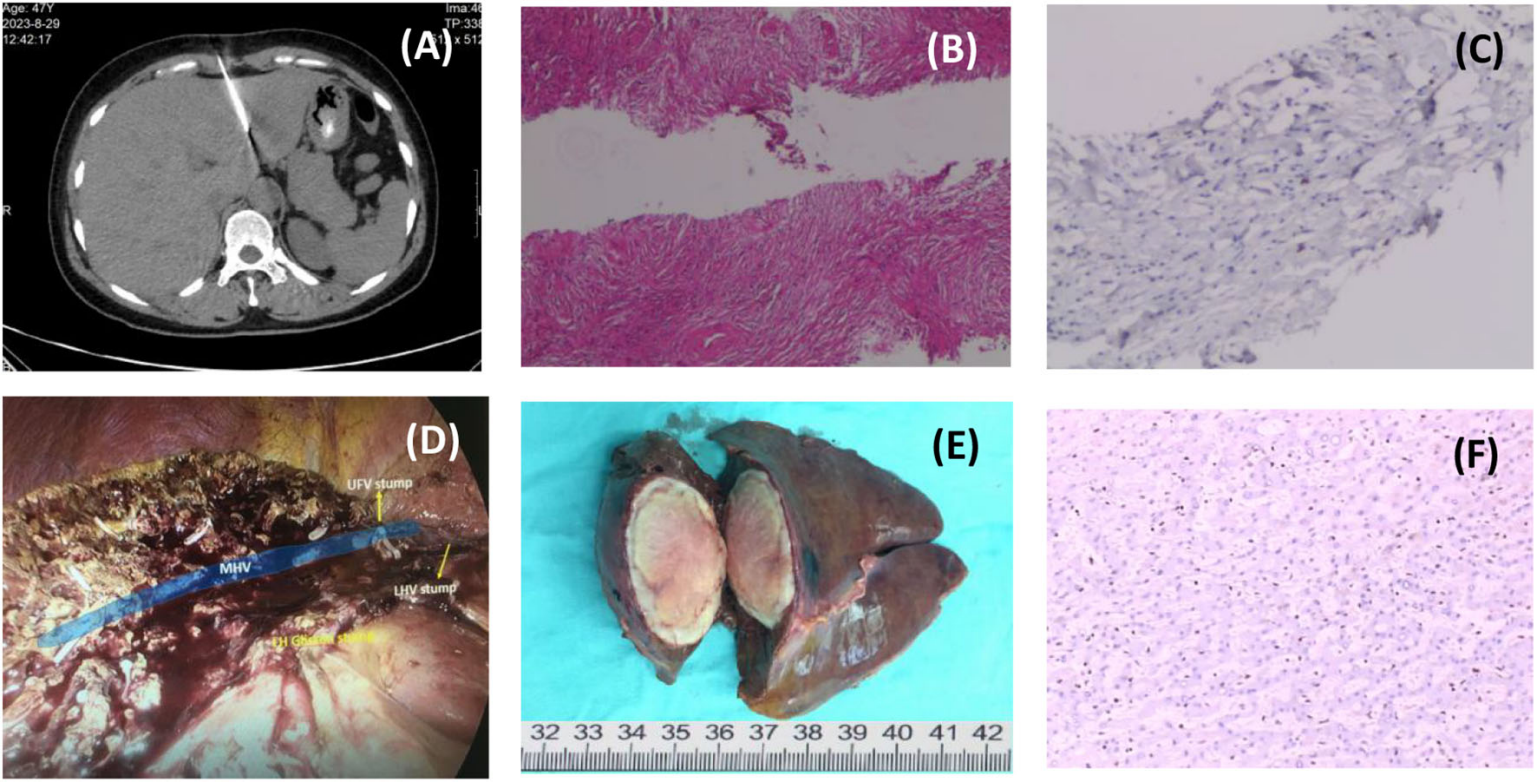
Process of treatment. [**(A)** Ct-guided needle biopsy of liver mass; **(B)** H&E staining of needle biopsies, 20×; **(C)** IHC staining of a needle biopsy, 200×; **(D)** Intraoperative photographs after tumor resection; **(E)** Surgical specimen; **(F)** IHC staining of postoperative pathology, 200×.].

The initial diagnosis was liver abscess, and CT-guided needle biopsy of the mass was performed while experimental antibiotic therapy (meropenem plus metronidazole) was administered(**Figure 2A**). Pathology showed a large number of collagen fibers with inflammatory cell infiltration but no pus (**Figure 2B, C**). Liver abscess was excluded after MDT discussion, and the patient was considered to be liver inflammatory myofibroblastic tumor (IMT) with fever. Because antibiotic therapy was ineffective, low-dose dexamethasone (5mg/d) was used to stabilize the body temperature, and a Laparoscopic left hemihepatectomy was performed (**Figure 2D, E**).

The pathological findings were unexpected. Microscopically, the tumor consisted of a large number of epithelioid spindle cell cords embedded in hyaline stroma, with mitotic figures, and a large amount of fibrous tissue with hyalinization around slit-like blood vessels (**Figure 2F**). Immunohistochemistry showed that SMA, Vimentin, SSTR2, CD31, TFE-3, ERG, FLI-1, D2-40 (podoplanin) were positive, and Ki67 positive index was about 10%. Based on the immunohistochemical results, it was considered that the tumor was derived from vascular endothelial cells, supplemented by molecular pathological examination showing WWTR1-CAMTA1 gene fusion. The final pathological diagnosis was hepatic epithelioid hemangioendothelioma (HEHE).

**Figure 3:**
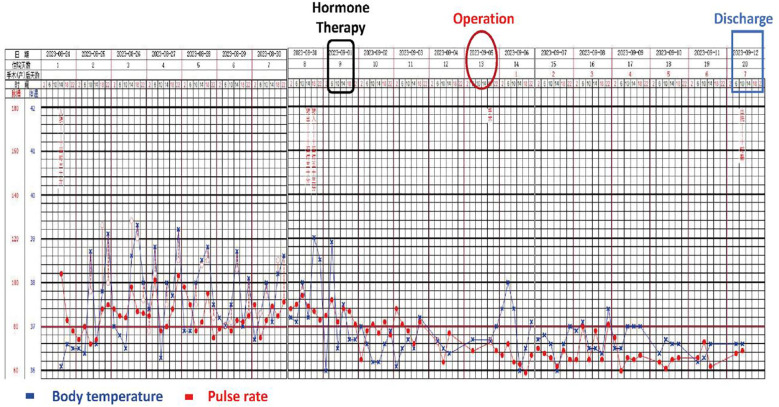
Trend plot of perioperative peak body temperature.

**Figure 4:**
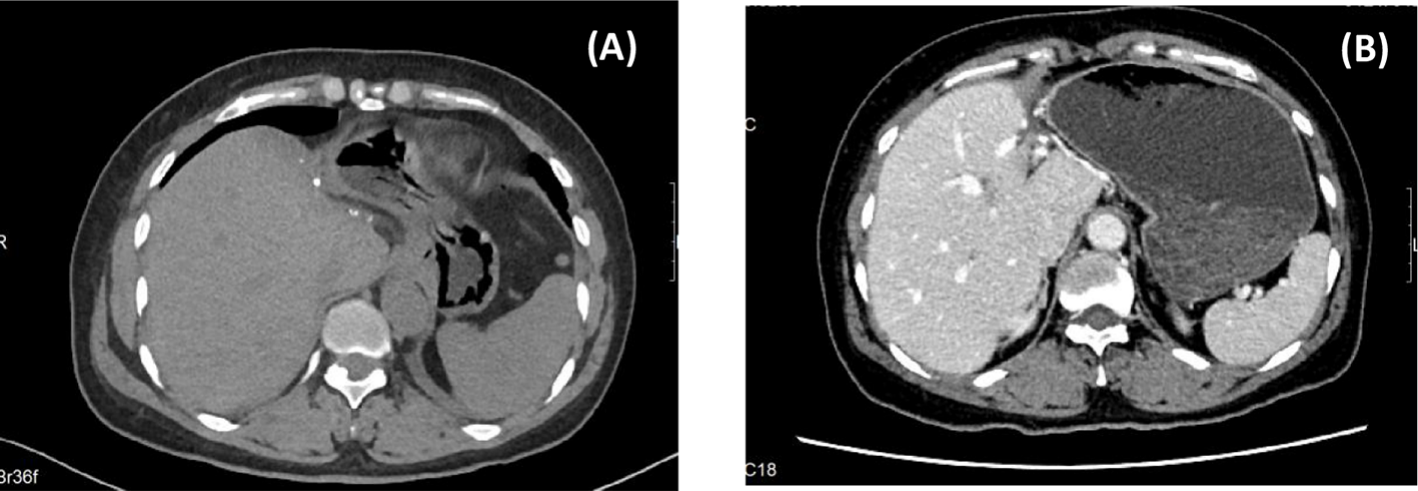
Postoperative FOLLOW-UP. [**(A)** CT images at 1 month after surgery; **(B)** CT images at 6 months after surgery.].

The patient recovered uneventfully without complications or fever and was discharged on POD 7 (**Figure 3**). Lenvatinib was administered orally to prevent metastasis. There was no recurrence after 6 months of follow-up (**Figure 4**).

## Discussion

Hepatic epithelioid hemangioendothelioma (HEHE) is a rare vascular tumor with biological behavior intermediate between benign hemangioma and malignant angiosarcoma. HEHE was first described by Weiss and Enzinger in 1982, and was classified as a malignant tumor by the World Health Organization(WHO) in 2013. Epidemiological surveys have shown that the incidence of HEHE is lower than 0.1-0.2/one million (1), and there is no obvious ethnic or regional clustering. HEHE is more common in middle-aged women, and the ratio of male to female is l:2.1. HEHE occurs most commonly in the liver and can show the characteristics of multicentric growth. About 20%-30% of the tumors can grow and migrate along the hepatic veins and sinusoid, and cause liver, lung and bone metastasis (2).

The exact etiology of HEHE is still unclear. Studies have shown that environmental factors such as long-term exposure to polyvinyl chloride (PVC), contraceptive pills or arsenic may be involved in the pathogenesis (3), but the evidence is not sufficient. Chronic liver inflammation, alcohol, and abnormal vascular stimulation are also considered to be involved in the pathogenesis of HEHE (3). Molecular pathological studies have revealed the key role of chromosomal translocations in the pathogenesis of HEHE, the most common being t (1; 3) (p36.3; q25) translocation, resulting in WWTR1-CAMTA1 gene fusion (about 90%). YAP1-TFE3 fusion gene was detected in a minority of cases (about 10%) (2). In addition, recent studies have shown that D2-40 (podoplanin) is highly expressed in HEHE (about 80% of cases are positive), suggesting its important value in the differential diagnosis of HEHE (4). Angiogenic factor signaling pathways, such as VEGF and FGF (5), are abnormally activated in HEHE, suggesting that tumor growth may depend on the angiogenic microenvironment. These molecular changes have become the molecular signatures of HEHE, which not only help to diagnose, but also provide potential targets for targeted therapy.

HEHE is a rare disease, and there is a lack of systematic retrospective studies with large data and specific diagnosis and treatment guidelines. Its clinical presentation is usually atypical or absent, or presents only as a liver mass. About 25% of patients are asymptomatic and are incidentally detected during physical examination (2). Laboratory tests and tumor markers may be completely normal, and even when the tumor is quite large, liver-function tests may be completely normal or show only mild elevations in alkaline phosphatase (ALP) and gamma-glutamyltransferase (GGT). Imaging examination often needs to be differentiated from a variety of liver solid tumors and liver abscess. Typical imaging changes include (6): (1) Color Doppler Ultrasound can show low echo in the center and rich blood flow signal in the periphery; (2) Contrast-enhanced ultrasound and enhanced CT showed characteristic “lollipop sign”: round-like masses without enhancement or with marginally enhanced edges, and the hepatic veins or portal veins can be seen running towards the lesion and terminating at the edge of the lesion. These two features together form a “lollipop sign” like image characteristic (7). (3) On MRI-T2WI, the “target sign” was observed: which consists of a low-density/low-signal nucleus surrounded by a layer of enhanced tissue and a thin surrounding low-density/low-signal halo, resembling a target ring (8). (4) Partial depression of the liver capsule, known as the “liver capsule retraction sign”, but it needs to be differentiated from the depression of the tumor capsule. PET-CT is of limited value in the diagnosis of HEHE, usually showing mild to moderate radioactivity concentration (SUVmax 2-5), which is only helpful in detecting extrahepatic metastases. Therefore, the diagnosis of HEHE must rely on histopathological examination and molecular pathology. The pathological diagnostic criteria of HEHE proposed by the WHO include (9): (1) Typical histological structure: epithelioid or dendritic tumor cells infiltrating hepatic sinusoids; (2) Characteristic cytological findings: cytoplasmic vacuolization (primitive vascular lumen) containing red blood cells; (3) Immunophenotype: at least 2 vascular endothelial markers positive (CD31/CD34/FVIIIRAg); (4) WWTR1-CAMTA1 or YAP1-TFE3 fusion gene was detected by molecular pathology.

According to the clinical manifestations and imaging features on admission, the first time it was misdiagnosed as liver abscess. After percutaneous liver biopsy, liver abscess was excluded, and it was misdiagnosed as Inflammatory myofibroblastic tumor(IMT) again according to the puncture pathological results. HEHE was finally diagnosed by immunohistochemistry and molecular pathology after surgery. We organized another MDT discussion after surgery and summarized the causes of misdiagnosis: (1) Misleading fever symptoms. Fever is very rare in HEHE. No case report of HEHE with fever as the main clinical manifestation has been retrieved, while fever is common in liver abscess and IMT. (2) IMT overlaps with HEHE in imaging manifestations, gross pathological features and immunohistochemical markers, which leads to difficulties in differential diagnosis. ① In terms of cellular origin, IMT is a benign or low-grade borderline tumor derived from mesenchymal tissue composed of myofibroblasts and inflammatory cells (10). ② In terms of imaging, it is difficult to distinguish the “target sign” of HEHE from the non-uniform and progressive enhancement of IMT. The imaging results of this case did not show the typical “target sign”, and the “lollipop sign” is easily confused with the signs of tumor compression. ③ In terms of cytopathological features, the main difference between them was the abundance of intracytoplasmic vascular lumen, which was a typical manifestation of intracytoplasmic vacuoles containing red blood cells in HEHE, and the abundance of spindle myofibroblasts in IMT. However, the amount of biopsy tissue was often not enough to determine the histocytological characteristics. Inflammatory cell infiltration in the HEHE tissue specimen of this case is extremely rare. It is often difficult to accurately observe the fine structure of cells and matrix components under the background of inflammation. ④ Immunohistochemically, both of them expressed endothelial cell markers CD31 and CD34 and myofibroblast marker SMA, but about 30% of HEHE expressed ERG, FLI-1, FVII-rag, CK (AE1/AE3) or EMA, and Caldesmon and ALK was more common in IMT (> 50%) (11). (3) Molecular pathological diagnosis becomes the key to the final differential diagnosis. The core driver mutations of HEHE were WWTR1-CAMTA1 fusion (about 90%) and YAP1-TFE3 fusion (10%), and few specific concomitant mutations were found. The core driver mutations of IMT are ALK rearrangements (70%), including TPM3-ALK (common) and RANBP2-ALK (highly aggressive), and are often accompanied by TP53 mutations (40%) and ROS1/PDGFRβ/NTRK (ALK-negative) fusion mutations (12, 13).

Another question worth further discussion is why febrile symptoms occur. After the postoperative MDT discussion, it was concluded that: (1) According to the perioperative peak temperature curve (**Figure 3**), the patient had fever for 4 months before the operation, but her body temperature quickly returned to normal after tumor resection, which proved that there was a direct relationship between tumor and fever. (2) Exogenous infection has been ruled out by preoperative etiological examination, experimental antibiotic treatment and monitoring of infection indicators. (3) No focal abscess, regional bile duct dilatation (tumor compression leading to obstructive cholangitis) and other physical factors that may cause fever were found on preoperative imaging. (4) Autoimmune disease spectrum examination to exclude immune-related factors; (5) Fever may be caused by two factors: ① Intratumoral inflammatory factors: Tumor biopsy and postoperative pathological results showed a large number of inflammatory cell infiltration and fibrous scar tissue in the tumor. Combined with the changes of serum CD3+/CD4+T cells, IL-6, IL-2 and CRP before and after surgery, it was speculated that fever was closely related to the internal inflammatory response of the tumor, that is, tumor fever caused by release of inflammatory mediators and absorbed heat caused by internal necrosis of the tumor. CD4+T cells, IL-6 and IL-2 may play a key role in mediating the persistent febrile symptoms. However, there is still a lack of direct evidence to prove the causal relationship between them, which needs to be further verified by single-cell sequencing and tumor immune microenvironment studies. ② Intratumoral high pressure factors: the scarring and high pressure in the tumor may cause hypoxia and necrosis in the tumor and the rapid influx of inflammatory mediators into the blood, which may eventually lead to frequent fever. (6) Is there intratumoral infection? Studies have shown that intratumoral bacterial colonization may protect tumors from the immune system and help them to spread and metastasizing in the body (14), but whether it causes long-lasting fever is still unknown. We have not performed further metagenomic analysis of tumor tissue to answer this question.

The treatment of HEHE should comprehensively consider the tumor range, growth rate, liver function status and general condition of patients, and adopt individualized strategies and MDT cooperation mode. Treatment methods include surgery (surgical resection or liver transplantation), local therapy (TACE, ablation, SBRT), systemic therapy, and targeted therapy. For patients with a single lesion or multiple lesions confined to a single liver lobe with sufficient residual liver function, surgery and R0 resection is the preferred treatment, with a 5-year survival rate of 70-80% and a 10-year survival rate of about 60% (9). According to the postoperative follow-up results, this case achieved R0 resection. At present, there is no clear guideline and consensus on the postoperative treatment of HEHE. For HEHE with high risk of recurrence, such as male, weight loss >10%, ascites, persistently elevated liver enzymes, high histological cell density, and high Ki-67 index (≥10%), oral targeted drugs(such as lenvatinib and lenalidomide) and lifelong follow-up can be considered (15). Of note, tumor recurrence and extrahepatic metastasis are not surgical contraindications and do not necessarily indicate a poor prognosis (16). Studies have found that the 5-year survival rate of patients with only small pulmonary nodules (<1cm) can still reach more than 60%. This unique biological behavior reflects the essential difference between HEHE and other malignant tumors.

## Conclusions

HEHE is a kind of low-grade malignant neoplastic lesions derived from vascular endothelial cells. HEHE with fever as the main feature is very rare, but not impossible. At this time, it should be carefully differentiated from IMT. Immunohistochemistry and molecular pathology are helpful for the final diagnosis.

## Data Availability

The original contributions presented in the study are included in the article/supplementary material. Further inquiries can be directed to the corresponding author.
